# Neural, behavioural and real-life correlates of social context sensitivity and social reward learning during interpersonal interactions in the schizophrenia spectrum

**DOI:** 10.1177/00048674211010327

**Published:** 2021-05-18

**Authors:** Esther Hanssen, Mariët van Buuren, Nienke Van Atteveldt, Imke LJ Lemmers-Jansen, Anne-Kathrin J Fett

**Affiliations:** 1 Department of Clinical, Neuro and Developmental Psychology, Faculty of Behavioural and Movement Sciences, and Institute for Brain and Behaviour (IBBA) Amsterdam, Vrije Universiteit Amsterdam, Amsterdam, The Netherlands; 2 CSI Lab, Institute of Psychiatry, Psychology and Neuroscience, Department of Psychosis Studies, King’s College London, London, UK; 3 Hersencentrum Mental Health Institute, Amsterdam, The Netherlands; 4 Department of Psychology, City, University of London, London, UK

**Keywords:** Functional magnetic resonance imaging, experience sampling method, trust, schizophrenia, social context processing

## Abstract

**Objective:**

Recent findings suggest that diminished processing of positive contextual information about others during interactions may contribute to social impairment in the schizophrenia spectrum. This could be due to general social context processing deficits or specific biases against positive information. We studied the impact of positive and negative social contextual information during social interactions using functional neuroimaging and probed whether these neural mechanisms were associated with real-life social functioning in schizophrenia spectrum disorders.

**Methods:**

Patients with a schizophrenia spectrum disorder (*N* = 23) and controls disorder (*N* = 25) played three multi-round trust games during functional magnetic resonance imaging scanning, with no, positive and negative information about the counterpart’s trustworthiness, while all counterparts were programmed to behave trustworthy. The main outcome variable was the height of the shared amount in the trust game, i.e. investment, representing an indication of trust. The first investment in the game was considered to be basic trust, since no behavioural feedback was given yet. We performed region-of-interest analyses and examined the association with real-life social functioning using the experience sampling method.

**Results:**

Social contextual information had no effect on patients’ first investments, whereas controls made the lowest investment after negative and the highest investments after positive contextual information was provided. Over trials, patients decreased investments, suggesting reduced social reward learning, whereas controls increased investments in response to behavioural feedback in the negative context. Patients engaged the dorsolateral prefrontal cortex less than controls during context presentation and showed reduced activity within the caudate during repayments. In patients, lower investments were associated with more time spent alone and social exclusion and lower caudate activation was marginally significantly associated with higher perceived social exclusion.

**Conclusion:**

The failure to adapt trust to positive and negative social contexts suggests that patients have a general insensitivity to prior social information, indicating top-down processing impairments. In addition, patients show reduced sensitivity to social reward, i.e. bottom-up processing deficits. Moreover, lower trust and lower neural activation were related to lower real-life social functioning. Together, these findings indicate that improving trust and social interactions in schizophrenia spectrum needs a multi-faceted approach that targets both mechanisms.

## Introduction

The ability to integrate social contextual information and behavioural feedback from others is necessary for successful social interactions (Ruz et al., 2011) and is an important foundation of trust in social relationships (Lewicki and Wiethoff, 2006). Individuals with a schizophrenia spectrum (SZ) diagnosis, hereafter referred to as patients, show a reduced ability in judging social signals (Penn et al., 2008). Previous research suggests two underlying mechanisms. First, there are deficits in learning from others’ behavioural feedback during social interactions, suggesting problems in bottom-up mechanisms. Second, there are problems with the integration of a priori contextual information in a top-down way (Chung et al., 2010; Hooker et al., 2011). Here, we investigate how these two mechanisms of social information processing impact on social behaviour in real-time interactions, using a modified neuroeconomic trust game (Fett et al., 2015).

Studies employing the trust game (Berg et al., 1995) in SZ have demonstrated that patients invested lower initial amounts, indicating lower trust towards others (Fett et al., 2012; Gromann et al., 2013; Lemmers-Jansen et al., 2018). In addition, SZ has been associated with a reduced ability to use others’ cooperative behavioural feedback to adjust trusting behaviour, i.e. bottom-up processing (Fett et al., 2012; Gromann et al., 2013). Research on the effects of social contextual information on trust in the general population demonstrated that trust increases in response to a trustworthy interaction partner, showing a strong impact of a priori information on trust in a top-down manner (Delgado et al., 2005). Patients, in contrast, show a diminished sensitivity to such prior positive information (Fett et al., 2012). The reduced ability to use bottom-up information and not being able to overcome distrust during positive interactions in response to such information, may explain real-life social impairment seen in SZ (Velthorst et al., 2016) and could be tackled with cognitive bias modification or other ways of cognitive remediation. However, it has not been investigated whether the insensitivity to social context reflects a more general processing deficit or a specific bias against positive social information.

Three core cognitive mechanisms have been suggested to underlie trust and decision-making in social contexts (Declerck et al., 2013). First, context processing and cognitive control, which are subserved by the dorsolateral prefrontal cortex (dlPFC) (MacDonald et al., 2000). Second, theory of mind, i.e. the ability to infer the mental states of others, in which medial prefrontal cortex (mPFC) and temporo-parietal junction (TPJ) are implicated as key regions (Carrington and Bailey, 2009; Schurz et al., 2014). Third, reward processing, which strongly involves the caudate nucleus (Krach et al., 2010; Sanfey, 2007).

Deficits in mentalizing, social reward processing (bottom-up) and social context processing (top-down) have been suggested to underlie lower trust, paranoia and social disconnection in SZ (Couture et al., 2006; Kapur et al., 2005; Velthorst et al., 2016). In SZ, prior studies have reported reduced activation within the dlPFC during context processing (Barch and Ceaser, 2012; Niendam et al., 2014), within the mPFC and TPJ during mentalizing (Green et al., 2015; Lee et al., 2004; Pinkham et al., 2008), and within the caudate during both non-social (Juckel et al., 2006b; Murray et al., 2008) and, more importantly, social reward processing (Fett et al., 2019; Gromann et al., 2013). This earlier work leads to the hypotheses that the dlPFC, mPFC, TPJ and caudate play an important role in disturbed social decision-making and context processing in SZ.

In this first-time investigation of the underlying mechanisms of disturbed trust and social interaction in SZ, we therefore probed the impact of different social contexts and investigated the underlying neural correlates in patients with SZ and healthy controls, using a modified version of an interactive trust game while measuring brain activity with fMRI. Since social interactions are embedded in peoples’ daily lives in a complex way, it is important to elucidate the association between the neural processes underlying social interactions and daily-life social engagement in SZ. To investigate this, we combined fMRI and the experience sampling method (ESM), a diary method (Delespaul, 1995). Initial ESM studies (Kluge et al., 2018; Moran et al., 2019) started to investigate how brain activation during task-based fMRI translates to real-world functioning. This method ensures high ecological validity because it allows for real-time monitoring of behaviour in daily-life contexts. In patients, Moran et al. (2019) found that greater hemodynamic signal change during (non-social) reward anticipation in caudate, insula and anterior cingulate was associated with greater anticipated pleasure and motivation for daily-life activities.

We hypothesized that (1) patients would show a general reduced sensitivity to prior information about the counterpart, reflected in no differences in baseline trust between conditions, i.e. first investments. Controls would increase investments from the negative to the positive context; (2) patients would not increase trust over trials in response to benevolent behavioural feedback, whereas controls would do so; (3) patients would engage the regions-of-interest (ROIs) to a lesser extent than controls: (a) in the left dlPFC during context presentation and investment (i.e. trusting) decisions, because of its specific role in context processing; (b) in the mPFC and right TPJ during context presentation and investment decisions, given their role in mentalizing mechanisms, which we expected to a greater extent in controls while processing prior social information compared to no information and (c) in the right caudate nucleus during the partner’s repayments (i.e. receipt of social reward); and (4) for patients, reduced trust and reduced activation in the ROIs during the trust game is associated with lower daily-life social functioning, i.e. more time spent alone, higher perceived social exclusion and lower perceived relationship quality, measured by ESM.

## Methods

### Subjects

Twenty-five patients with an SZ diagnosis and 26 controls without a personal or family history of SZ were included (for recruitment, see Supplement – A). Inclusion criteria were (1) age 18–65 years, (2) good understanding of the English language and (3) intelligence quotient (IQ) >70. An additional criterion for patients was an SZ diagnosis according to the *International Statistical Classification of Diseases and Related Health Problems–Tenth Edition* (ICD-10; World Health Organization (WHO), 1992), which was confirmed with the treating National Health Service (NHS) clinician. Exclusion criteria were (1) a history of any neurological conditions and (2) a diagnosis of alcohol/drug dependence within 6 months. One control subject did not complete MRI scanning due to anxiety. Two patients were excluded from analyses due to excessive movement (framewise displacement ⩾1.5 mm in ⩾20% of the volumes per run). Therefore, analyses were performed on 23 patients and 25 controls. Forty-four participants completed the ESM measurements (20 patients and 24 controls). The London – Harrow Research Ethics Committee (14/LO/0071) approved this study.

### Measures

#### Estimated cognitive ability

To assess an estimated cognitive ability, an abbreviated two-test version of the Wechsler Abbreviated Scale of Intelligence (WASI) was used (Wechsler, 1999), which consisted of the vocabulary subtest and the matrix reasoning subtest. WASI scores are reported in Table 1.

**Table 1.** Participant demographics and patient clinical characteristics.

	Controls (*N* = 25)	Patients (*N* = 23)	Statistic	*p*-value
Age – M (SD)	36.02 (7.34)	39.86 (9.10)	β = 0.23	0.11
Gender (% male)	68.0	82.6	χ^2^(1) = 1.36	0.24
IQ – M (SD)	116.68 (10.18)	98.30 (11.80)	β = −0.64	**<0.0001**
Diagnoses (%)				
Schizophrenia		73.9		
Schizoaffective disorder		17.4		
Psychotic disorder		8.7		
Medication (%)				
Atypical antipsychotics		82.6		
Typical antipsychotics		13.0		
None		4.4		
PANSS – M (SD)				
Negative scale		1.75 (0.45)		
Positive scale		2.11 (0.86)		
Amotivation factor		1.92 (1.15)		
Diminished expression factor		2.45 (1.36)		
P6 (suspiciousness)		2.70 (1.23)		
Experience sampling (ESM)				
Alone time (%)	55	71	β = 0.31	**0.04**
Social exclusion – M (SD)	1.77 (0.94)	2.64 (0.81)	β = 0.45	**0.002**
Relationship quality – M (SD)	5.38 (1.12)	5.18 (0.86)	β = −0.10	0.53

M: mean; SD: standard deviation; IQ: intelligence quotient; PANSS: Positive and Negative Syndrome Scale; ESM: experience sampling method. Statistically significant differences are bold faced.

#### Symptoms

The Positive and Negative Syndrome Scale (PANSS) semi-structured interview was used to measure symptom severity in the 2 weeks prior to testing in patients (Kay et al., 1987). Fourteen items evaluate the severity of positive and negative symptoms (1 = absent to 7 = extreme). PANSS scores are reported in Table 1.

#### Trust game

**Figure 1.**
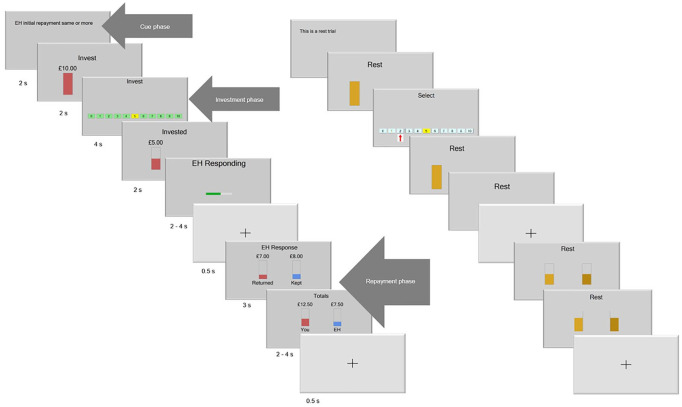
Left: investment trial with positive context (1) context cue: ‘initial repayment same or more’, (2) investment cue, (3) investment choice, made by scrolling over a horizontal bar ranging from £0 to £10, which started at £5, (4) invested amount displayed, (5) wait for game partners’ response (jittered), (6) fixation cross, (7) returned amount displayed, (8) round totals (kept and given amount added) for both players (jittered) and (9) fixation cross. Right: control trial, similar to the experimental trials, except for (a) ‘Invest’ was replaced by ‘Rest’ and (b) in the investment phase, participants had to move the cursor to the marked number.

To measure the impact of social context processing in social interactions, we employed a modified multi-round trust game (Berg et al., 1995; Gromann et al., 2013; King-Casas et al., 2005). In a multi-round classic trust game, the first player, i.e. the investor, is given an initial endowment of £10 and has to invest a chosen amount between zero and ten pounds. This amount is tripled and given to the second player, i.e. the trustee. The trustee then decides whether and how much of the tripled amount he or she wants to give back to the investor. The chosen amount to invest by the investor reflects trust (given that the trustee can choose not to return any money). In this study, participants played the role of the investor and played the trust game three times, with three different hypothetical counterparts. In one game (condition), the trust game was presented without prior social contextual information, i.e. a classic multi-round trust game, while the other two conditions were modified to examine social context processing. In the negative and positive context conditions, participants first played three ‘blind’ rounds, without seeing the repayments of the interaction partner. These rounds were implemented to establish the cooperativeness of the trustee (Fett et al., 2012). When making their blind investments, participants saw the following the message: ‘Determining E.H.’s initial average returns’. After these three ‘blind’ investments, they viewed the following message on the screen pointing out the average returns (more or less than invested) of the trustee during the blind investments (i.e. the social context): ‘On average your partner E.H. returned more/less than you invested’. This message was shown before each trial in the trust game. Participants completed a total of 120 trials (60 experimental and 60 control), equally divided over the three conditions (no context, positive context and negative context). Control trials were included to control for general effects of motor and visual activation elicited by the task. An experimental and control trial with the respective phases and timings is displayed in Figure 1.

Participants were instructed that they played with a real human player via the Internet but were actually playing with a pre-programmed computer that behaved in the same benevolent probabilistic manner in all three contexts (for algorithm, see Supplement – B). After completion of the trust game, participants were asked whether they thought the other players were real and trustworthy, on a 7-point Likert-type scale. Ratings on whether the other players were unreal did not differ between groups (16% controls and 13% patients, *p* > 0.83), and were unrelated to investments in the trust game (*p* > 0.82). Also, the ratings of trustworthiness of the interacting partners did not differ between groups (*p* > 0.59).

#### ESM – measurement of social engagement

ESM (Palmier et al., 2011), a structured diary technique, was used to measure social engagement in daily life. The ESM device (iPod) gave a signal to fill in the questionnaire 10 times a day, by means of a pseudo-random ‘beep’ on 7 consecutive days. We included several questions to probe real-life social functioning in terms of social engagement, social exclusion and quality of social relationships. The question that was used assessing social engagement was ‘*Are you alone?*’ (yes/no). Perceived social exclusion was assessed when individuals were alone using an average of the two items: ‘*I feel lonely*’ and ‘*I feel excluded*’ (Cronbach’s α = 0.82). The perceived quality of social relationships was assessed when individuals were in social company with an average of the four items: ‘*I like the person(s) I am with*’, ‘*I feel close to them*’, ‘*They are dependable*’ and ‘*I trust them*’ (Cronbach’s α = 0.90). ESM items were rated on a 7-point Likert-type scale.

### Procedure

The two sessions took place at the Institute of Psychiatry, Psychology and Neuroscience (IoPPN), King’s College London. Participants gave written informed consent before the study.

Participants first completed a demographic questionnaire and several practice trials before playing the trust game in the MRI scanner. They were told that they would receive the earnings from one randomly selected round to keep them motivated (between £0 and £30). For fairness reasons, all participants received a payment of £5. After the trust game, they completed the questionnaire on their perception of the game partners. Next, the PANSS interview was administered. Finally, an explanation of the iPod was given to participants and they completed one practice questionnaire. The morning after the first session the 7 days ESM data collection started. All participants were contacted by phone on day 2 for guidance in case of any problems or difficulties with the iPod. The second session consisted of assessment of the WASI subtests. Participants handed in the iPod and experiences were discussed. At the end of the study, each participant received payment (£60 + £5) for participation.

### Statistical analyses

#### Behavioural analyses

Statistical analyses were performed using STATA version 14 (StataCorp, 2015). We examined group differences in demographics using chi-square tests and regression analyses. Investments were analysed using mixed effects multilevel random regression analyses (MIXED) to account for repeated measurements within persons, with (first) investment as dependent variables and with group (control and patient) and context (negative, no and positive) and their respective interactions as independent variables. For investments over trials, trial number (1–20) was added to the model. Mixed effects multilevel random regression analyses (MIXED) were used to examine associations between investments and ESM indices of real-life social functioning (% alone, social exclusion and quality of social relationships) across contexts. Interactions were probed with the CONTRAST command. Analyses with a significant group effect are additionally reported with estimated IQ as a covariate. Additional analyses on association with symptoms are reported in Supplement – E.

#### FMRI data acquisition and scanning parameters

Imaging data were acquired using a 3T GE Signa Neuro-optimized magnetic resonance (MR) System at the Centre of Neuroimaging Science of the IoPPN. Functional images were acquired by a T2*-weighted echo-planar imaging sequence scanning 39 axial slices of 3.0 mm thick with 0.3 mm gap. The in-plane resolution was 3.3 mm × 3.3 mm (FOV 211 × 211), flip angle = 75°, TR = 2.00 seconds and TE = 30 ms. There were 413 volumes per run. For anatomical reference, a T1-weighted image (196 slices; isotropic voxels of 1.2 mm; TR = 7.312 ms; TE = 3.016 ms; flip angle = 11°; FOV = 270 mm) was acquired.

Imaging data were analysed using Statistical Parametric Mapping 12. Pre-processing of the functional images consisted of realign and unwarp, and coregistration to individual anatomical images. Next, using unified segmentation, anatomical images were segmented and normalization parameters were estimated. These parameters were used to transform functional and anatomical images to a Montreal Neurological Institute (MNI) template. Subsequently, smoothing was applied (Gaussian kernel 6 mm full-width at half-maximum), and the last three volumes were removed at the ending of the task.

#### ROI analyses

A general linear model was used per run in which the three phases in the game were modelled as regressors of interest for the experimental and control condition separately (see Figure 1). The cue phase was time-locked to the start of each trial (duration 2 seconds), the investment phase started after the investment cue (duration 4 seconds) and the repayment phase started at the beginning of the repayments shown and was modelled until the end of the displayed totals (duration 5–8 seconds). All other game phases were combined into one regressor of no interest (the investment cue, the invested amounts, waiting for the partners’ response and the two fixation crosses). All phases were modelled using a box-car function convolved with the hemodynamic response function (Friston et al., 1995). To correct for motion, the six realignment parameters and regressors for volumes detected as motion-corrupted, calculated by DVARS metric as implemented in FSL, version 6.00 (FMRIB’s Software Library, www.fmrib.ox.ac.uk/fsl) by FSL Motion Outliers (https://fsl.fmrib.ox.ac.uk/fsl/fslwiki/FSLMotionOutliers) were included in the design matrix, making the total number of regressors in the model variable for each individual, with a minimum of 10 regressors (cue phase, investment phase, repayment phase, regressor of no interest and six motion parameters). A high-pass filter of 128 seconds was used. Subsequently, for each phase of interest, contrast images were created by contrasting a specific phase of the experimental condition with the corresponding phase in the control condition.

A priori ROI analyses were performed. Talairach coordinates were converted to MNI space (https://bioimagesuiteweb.github.io/webapp/mni2tal.html), resulting in the following ROI MNI coordinates: right caudate (17, 20, 3), right TPJ (50, −56, 27), mPFC (−3, 64, 24) (Gromann et al., 2014) and the left dlPFC (−43, 18, 29) (MacDonald and Carter, 2003). ROIs were created in MarsBaR with an 8-mm sphere (version 0.44; http://marsbar.sourceforge.net). For each ROI and each subject, average signal change (beta estimate) was extracted to investigate group and context effects, and to test association between the fMRI and the ESM data. Additional analyses on association with symptoms (PANSS positive scale, PANSS negative scale, PANSS suspiciousness, PANSS amotivation factor and PANSS diminished expression factor; Supplement – E) and IQ are reported. Mixed effects multilevel random regression analyses (MIXED) were used to examine associations between ROI beta estimates and ESM indices of real-life social functioning (% alone, social exclusion and quality of social relationships) across contexts. The results of the ROI-based analyses were Bonferroni corrected at α levels of 0.0125 per test (0.05/4, as tests were performed with data from four ROIs).

#### Exploratory analyses

We also performed exploratory whole-brain analyses, investigating neural activation beyond the predefined ROIs for all three game phases. Analyses were corrected at family-wise-error (FWE) whole-brain cluster significance threshold of *p* = 0.05 (see Supplement – C).

## Results

### Behavioural analysis

Groups did not differ in age and gender. Patients had a lower estimated cognitive ability than controls. Percentage of time spent alone was higher in patients than controls. Patients felt lonelier and more excluded, but reported a similar quality of their social relationships compared to controls (see Table 1).

#### Baseline trust: context effect and group differences in first investments

First investments were examined to establish context effects on baseline trust (Table 2). There was a significant group-by-context interaction (χ^2^(2) = 7.34, *p* = 0.02) which remained significant when estimated IQ was added to the model (χ^2^(2) = 7.34, *p* = 0.02). Estimated IQ was not significantly associated with baseline trust (*p* = 0.32). The context effect was only significant in controls, who made lower investments in the negative than the no context condition (*b* = −1.28, 95% confidence interval CI = [−2.23, −0.33], *p* = 0.009) and higher investments in the positive than the no context condition (*b* = 1.32, 95% CI = [0.35, 2.28], *p* = 0.007). Patients’ first investments did not differ by context (both *p* > 0.75). In all three contexts, first investments did not differ significantly between groups (all *p* > 0.07).

**Table 2.** Task performance – first investments in GBP (£).

	Negative context, mean (SD)	No context, mean (SD)	Positive context, mean (SD)	Context difference	Statistic	*p*	95% CI
Controls (*N* = 25)	5.00 (2.91)	6.28 (2.25)	7.60 (2.04)	Negative < no Positive > no	−1.28 1.32	**<0.01** <**0.01**	[−2.24, −0.32] [0.36, 2.28]
Patients (*N* = 23)	6.35 (2.90)	6.48 (2.74)	6.70 (2.93)	Negative = no Positive = no		*p* = 0.85 *p* = 0.75	

SD: standard deviation; CI: confidence interval. Statistically significant differences are bold faced (p<0.01).

#### Changes in trust over trials: context effect and group differences

**Figure 2.**
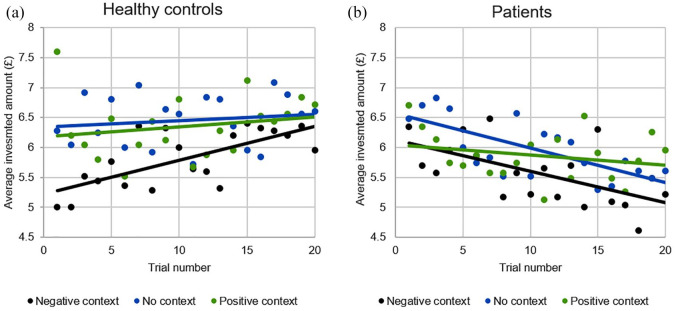
Investments over trials in (a) controls and (b) patients, showing investments in the negative, no and positive context conditions.

We examined interference of context information on changes in trust (i.e. investments) over time (Figure 2(a) and (b)). The three-way interaction between group, context and trial was marginally significant (χ^2^(2) = 5.07, *p* = 0.07), and analyses by group showed a marginally significant interaction of context-by-trial number in controls (χ^2^(2) = 5.26, *p* = 0.07) but not patients (*p* = 0.21). Across trials, both groups showed a context effect with significant differences between negative and no contexts, which was larger in controls than patients (controls: *b* = −0.64, 95% CI = [−0.88, −0.39], *p* < 0.0001 and patients: *b* = −0.39, 95% CI = [−0.67, −0.11], *p* = 0.006), but no differences between the positive and no contexts (both *p* > 0.41). Both groups showed a significant main effect of trial number; controls increased investments over time, while patients decreased their investments (controls: *b* = 0.03, 95% CI = [0.01, 0.04], *p* = 0.002 and patients: *b* = −0.04, 95% CI = [−0.06, −0.02], *p* < 0.0001).

In addition, analyses by context showed that the effect of trial number was most pronounced and in opposite directions in patients and controls in the negative context (group-by-trial number interaction (χ^2^(2) = 21.21, *p* < 0.0001), controls: *b* = 0.06, 95% CI = [0.03, 0.8], *p* < 0.001 and patients: *b* = −0.05, 95% CI = [−0.08, −0.02], *p* < 0.001). In the no context condition, there was a group-by-trial number interaction (χ^2^(2) = 8.37, *p* = 0.004); no significant changes in investments were found controls (*p* = 0.51), but patients invested significantly less over time (*b* = −0.06, 95% CI = [−0.10, −0.01], *p* = 0.02). Groups did not differ significantly in the positive context condition and did not show investment changes over trials (all *p* > 0.30). Additional analyses between symptoms and behavioural results are reported in Supplement – E.

### fMRI analysis

#### ROI analyses by trust game phase

##### Cue phase

**Figure 3.**
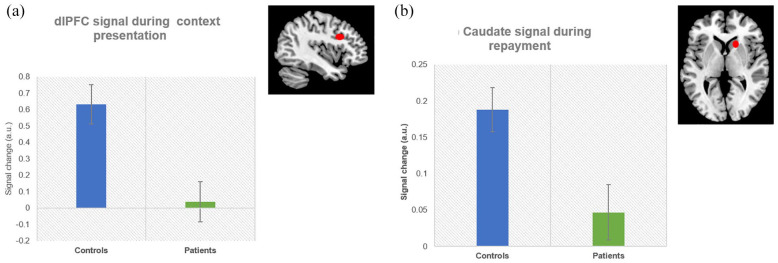
Signal change (in arbitrary units) in (a) the dlPFC during the cue phase (i.e. social context presentation) and (b) the caudate during repayment (i.e. receipt of reward) in controls and patients. Error bars depict the standard errors.

There were no significant group-by-context interactions in any ROIs (all *p* > 0.12). A significant group effect in the left dlPFC showed lower activation in patients than controls (*b* = −0.59, 95% CI = [−1.00, −0.18], *p* = 0.004, Figure 3(a)). The effect remained significant when the IQ estimate was added to the model as covariate (*b* = −0.71, 95% CI = [−1.24, −0.17], *p* = 0.009). The IQ estimate was not significantly related to left dlPFC activation (*p* = 0.51).

##### Investment phase

There were no significant group-by-context interactions (all *p* > 0.08), nor main effects of group (all *p* > 0.50) or context in any of the ROIs (all *p* > 0.11).

##### Repayment phase

There were no significant group-by-context interactions or context effects for the right caudate or mPFC. A significant group effect showed lower right caudate activation in patients compared to controls (*b* = −0.14, 95% CI = [−0.25, −0.03], *p* = 0.01, Figure 3(b)). This effect remained significant when the IQ estimate was added to the model as covariate (*b* = −0.16, 95% CI = [−0.30, 0.004], *p* = 0.028). The IQ estimate was not significantly related to right caudate activation (*p* = 0.66). Higher mPFC activation was found in patients than controls (*b* = 0.32, 95% CI = [0.003, 0.62], *p* = 0.047). However, this result did not survive Bonferroni correction. There were no interaction, group or context effects in any other ROI (all *p* > 0.16).

Given that we observed a behavioural group difference in investments over trials, we performed exploratory analyses probing the change in ROI activation over investment trials. The results are reported in Supplement – D. Additional analyses between symptoms and ROI results are reported in Supplement – E.

#### Associations between investment, ROI activation and real-life social functioning in patients

We were specifically interested to examine whether lower neural activation within the left dlPFC and the right caudate found in patients was related to the level of real-world social functioning. In addition, we explored this association in the less robustly increased mPFC signal in patients.

Across contexts, higher investments were significantly associated with less time spent being alone (*b* = −0.02, 95% CI = [−0.05, −0.001], *p* = 0.04). A significant interaction between context and perceived relationship quality on investments (*X*(2) = 23.01, *p* < 0.0001) showed trend-level associations between higher relationship quality and higher investments in the positive (*b* = 0.85, 95% CI = [−0.06, 1.78], *p* = 0.07) and negative (*b* = 0.94, 95% CI = [−0.06, 1.78], *p* = 0.06) conditions, but not the no context condition (*p* = 0.79). There was a significant interaction between context and social exclusion on investments (*X*(2) = 13.19, *p* < 0.0001), indicating an association between lower perceived social exclusion and higher investments the no context condition only (*b* = −1.17, 95% CI = [−1.97, −0.35], *p* = 0.005, other *p* *>* 0.23).

There were no significant associations between any measure of real-life social functioning and dlPFC activation (all *p* > 0.21). A significant interaction between social exclusion and context emerged for the right caudate (*X*(2) = 8.51, *p* = 0.01); lower perceived social exclusion was marginally significantly associated with higher caudate activation in the positive context only (*b* = −0.17, 95% CI = [−0.36, −0.018], *p* = 0.07, all other *p* > 0.18). No significant associations were present with social relationship quality or time spent alone (both *p* > 0.17). Higher perceived social relationship quality was associated with higher mPFC activation (*b* = 0.27, 95% CI = [0.002, 0.54], *p* = 0.048). There were no significant associations between mPFC activation and social exclusion or time spent alone (both *p* *>* 0.22).

## Discussion

Using a novel, modified trust game, we examined the impact of social context on trust and social reward and the underlying neural activation patterns during real-time social interactions. In addition, we probed the associations with real-life social functioning in SZ. Patients showed no differential effect of social context on first investments, regardless of the valence or the absence of a context, whereas controls showed the expected distinct context effect with highest levels of trust in response to positive and lowest levels of trust in response to negative social information. This suggests a general insensitivity to social context instead of a bias against positive social contextual information in patients. Patients also did not increase trust in response to benevolent behavioural feedback whereas controls did. Within the patient group, the findings indicate an association between lower trust and lower real-world social functioning.

We found overall lower activation in the left dlPFC during context presentation and less engagement of the right caudate nucleus during repayments in patients compared to controls. These results suggest that SZ is associated with a general insensitivity to social contexts and with a reduced sensitivity to social reward. On the neural level, we also found an association between caudate activation and lower real-life social functioning in patients.

### Social context effect on baseline trust

For baseline trust (i.e. the first investment where partner feedback has not yet been received), we found reduced sensitivity to positive and negative social contextual information in patients compared to controls. This seems to reflect a general insensitivity to social contextual information. These results strengthen and extend previous evidence of a social context top-down processing deficit in SZ (Baez et al., 2013; Fett et al., 2012; Niendam et al., 2014); however, this may not generalize to tasks concerning different types of information processing. This insensitivity points to persistent a priori beliefs about other people in SZ. It is important to unravel whether this context sensitivity is a risk factor for developing an SZ disorder or secondary to the disorder or related factors, by examining whether this insensitivity is also found in first-episode patients and individuals at high risk for SZ. In this study, patients tended to approach social interactions with similar trusting behaviour as controls, in line with findings in first-episode patients (Fett et al., 2019), but contrasting results have also been found in chronic and first-episode patients and individuals at clinical high risk (Fett et al., 2012; Gromann et al., 2013; Lemmers-Jansen et al., 2018).

### Trust over time: the effects of benevolent feedback

As hypothesized, patients did not increase trust in response to benevolent partner feedback, in line with previous literature in patients with chronic psychosis (Fett et al., 2012; Gromann et al., 2013). Controls, however, increased trust in the negative context and showed stable levels of trust after no and positive information, suggesting that prior positive beliefs about others were matched by the benevolent partner feedback. Patients seem to have difficulties to overcome the given prior negative information, i.e. persistent a priori negative beliefs about others. Patients showed a tendency to reduce trust, even though subjective ratings of trustworthiness of the interacting partner were similar for both groups. Other studies have shown that patients show reduced sensitivity to social rewards, such as smiles (Catalano et al., 2018). Our results strengthen the evidence that patients have deficits in bottom-up processing of partner feedback, which might be due to an insensitivity to social reward (Fett et al., 2012; Gromann et al., 2013). The reduced ability to increase trust could explain patients’ reduced motivation to engage in social behaviour (Krach et al., 2010).

### Neural findings during social interactions with contextual information

In support of our hypotheses, we found reduced left dlPFC activation in patients compared to controls during social context presentation. The dlPFC has been implicated in deficits in non-social context processing in schizophrenia (Barch and Ceaser, 2012; Niendam et al., 2014) and is viewed as key region in top-down cognitive control (MacDonald et al., 2000). The impairment in top-down modulation of trust in response to contextual information may be related to reduced engagement of the dlPFC in SZ. This result that dlPFC activation was lower in all contexts supports a general insensitivity rather than a specific bias. We did not see any group differences in ROI activation during investments. However, during the repayment phase, where reward processing takes place, we found context independent blunted activation in the right caudate nucleus in patients compared to controls. The caudate is a highly innervated by dopaminergic neurons (Björklund and Dunnett, 2007). Aberrant regulation of dopamine is thought to play a key role in SZ and reward processing (Howes and Kapur, 2009), and may account for the insensitivity to social feedback, i.e. reward. This finding adds to evidence showing deficits in social reward processing in SZ (Gromann et al., 2013; Lee et al., 2018) and may explain why patients fail to increase trust over time. A less robust effect was found in the mPFC; activation was higher in patients compared to controls. This was unexpected, but may be related to the role of the mPFC in reward-based action selection (Euston et al., 2012). We speculate that this may point to a compensation mechanism for reduced engagement of the caudate, which is tentatively supported by the correlations with real-world social outcomes, which are discussed in the following section.

### Associations with real-life social engagement

Previous social neuroscience studies have started to investigate the link between the reward related processing in the brain and daily life (Bakker et al., 2019; Moran et al., 2019), yet associations with real-life social functioning have not been considered in schizophrenia, despite yielding meaningful insights in healthy controls (Bickart et al., 2011; Kanai et al., 2012; Lewis et al., 2011). We found that in real life, patients spent more time alone than healthy controls, in line with previous work (Pinkham and Penn, 2006; Velthorst et al., 2016). They reported higher feelings of loneliness and social exclusion, but a similarly good relationship quality as controls. On the behavioural level, higher investments were associated with higher social functioning in real life. A higher level of trust may create an advantageous basis for engaging in meaningful social relationships (Campellone et al., 2016). This could positively impact on social support networks, which in turn could aid in recovery (Corrigan and Phelan, 2004). At the neural level, we found that in patients, lower activation in the caudate in the positive context was marginally significantly associated with higher perceived social exclusion. More engagement of this key reward area may be related to heightened experience of positive social interaction, i.e. social reward (Gromann et al., 2013). This could lead to a higher sense of belonging or inclusion in social relationships. In addition, higher activation in the mPFC was associated with higher reported relationship quality in daily life, which would support the role of mentalizing abilities (Schurz et al., 2014). However, it is possible that this finding also relates to higher feelings of (social) reward (Euston et al., 2012). Future evidence from larger replication studies will be needed to strengthen and support these initial results.

### Limitations

The current results should be interpreted in light of the following limitations. First, this study is an initial investigation of social context processing in relation to neural activation and in relation to daily-life social functioning in a relatively small sample of relatively stable patients with an SZ diagnosis. Specifically, our patient sample had relatively low symptom levels: negative and positive scores of, respectively, 14.74 (standard deviation (SD) = 6.15) and 12.26 (SD = 3.25). We defined these scores as relatively low, since, with respect to the 7-point Likert-type scale of the PANSS items and the number of items in the positive and negative subscale, the item scores of 1 and 2 are, respectively, absent and minimal (Kay et al., 1987), (see also Supplement – E). Consequently, our results need to be interpreted with caution when it comes to generalizability and larger replication studies are warranted before any firm conclusions can be made. Second, medication type has been found to have an effect on reward processing in SZ (Juckel et al., 2006a); however, the majority of participants in our sample were on atypical antipsychotics (see Table 1), which are thought to normalize reward processing (Nielsen et al., 2012; Schlagenhauf et al., 2008). In addition, healthy first-degree relatives of patients with SZ show reduced social reward processing, without any medication confounds (Gromann et al., 2014; Hanssen et al., 2020). Therefore, the current findings are not likely to represent an enhancement of the effects; they may even reflect an underestimation, compared to expected effects in unmedicated patients. Studying unmedicated patients is valuable, but poses a great challenge due to the clinical reality. Third, subjective self-report measures, like ESM, may raise the question of response accuracy or social desirable, which may be related to suspiciousness. However, ESM is a widely used and well-validated method in psychiatric and schizophrenia samples (Delespaul, 1995; Myin-Germeys et al., 2018) and has the advantage that it does not rely on retrospective recall (Potheegadoo et al., 2012). Moreover, our sample showed a good compliance and did not report any issues related to the use of the ESM app during the debriefing. We therefore have no grounds to assume that responses were inaccurate. Finally, not all participants believed they were playing with a real human player; however, this was not associated with investments and adequate changes in investments in controls after context information and behavioural feedback indicated that the experimental manipulation was effective.

### Concluding remarks

This is the first study to investigate the neural correlates of social context processing and the link with real-life social functioning in SZ. We provide evidence that patients do not modulate their behaviour in response to social context information (i.e. top-down processing) and positive behavioural cues of others (i.e. bottom-up processing). Our results point to a general reduced sensitivity to social information on a behavioural and neural level. The findings suggest that increasing trust may facilitate social engagement in patients. Also, indices of real-life social functioning seem to be associated with lower neural activity in reward- and context-processing (i.e. cognitive control) brain areas. This study suggests that improving social interaction in SZ requires a multi-faceted approach in clinical practice, which considers both bottom-up and top-down processing of social information. An example of an intervention that incorporates these two facets is the Social Cognition and Interaction Training (SCIT) (Penn et al., 2007), targeting social skills bottom-up and social cognition top-down, which shows promising effects on social functioning. Clinical practice will benefit from newly developed treatments, building on the SCIT for instance, targeting these facets in daily life by implementing an ecological momentary intervention by means of a smartphone-based treatment. In addition, future studies are warranted to investigate how this is related to fine-grained assessments of functional capacity and real-world social functioning.

## Supplemental Material

sj-docx-1-anp-10.1177_00048674211010327 – Supplemental material for Neural, behavioural and real-life correlates of social context sensitivity and social reward learning during interpersonal interactions in the schizophrenia spectrum Click here for additional data file. Supplemental material, sj-docx-1-anp-10.1177_00048674211010327 for Neural, behavioural and real-life correlates of social context sensitivity and social reward learning during interpersonal interactions in the schizophrenia spectrum by Esther Hanssen, Mariët van Buuren, Nienke Van Atteveldt, Imke LJ Lemmers-Jansen and Anne-Kathrin J Fett in Australian & New Zealand Journal of Psychiatry
