# Supplementary material for: Neural, behavioural and real-life correlates of social context sensitivity and social reward learning during interpersonal interactions in the schizophrenia spectrum
Source: Aust N Z J Psychiatry. 2021 May 18;56(1):59–70. doi: 10.1177/00048674211010327 (PMC8721616; doi:10.1177/00048674211010327)
Supplement: sj-docx-1-anp-10.1177_00048674211010327 – Supplemental material for Neural, behavioural and real-life correlates of social context sensitivity and social reward learning during interpersonal interactions in the schizophrenia spectrum [file sj-docx-1-anp-10.1177_00048674211010327.docx]

**Supplementary material**

**Neural, behavioural and real-life correlates of social context sensitivity and social reward learning during interpersonal interactions in the schizophrenia spectrum**

Hanssen, E. ^1,2,3^, van Buuren, M. ^1^, Van Atteveldt, N. ^1^, Lemmers-Jansen, I.L.J. ^1,2^, Fett, A.-K.J. ^1,2,4^

^1^ Department of Clinical, Neuro and Developmental Psychology, Faculty of Behavioural and

Movement Sciences, and Institute for Brain and Behaviour (IBBA) Amsterdam, Vrije Universiteit Amsterdam, the Netherlands
^2^ CSI Lab, Institute of Psychiatry, Psychology and Neuroscience, Department of Psychosis Studies, King’s College London, London, United Kingdom

^3^ Hersencentrum Mental Health Institute, Amsterdam, the Netherlands

^4^ Department of Psychology, City, University of London, London, United Kingdom

* Corresponding author: Esther Hanssen

Postal address: Vrije Universiteit Amsterdam, Faculty of Behavioural and Movement Sciences, Van der Boechorststraat 7, 1081 BT Amsterdam

Email address: emehanssen@gmail.com

***Supplement A:*** *Recruitment patients and healthy controls*

The patient group was recruited via the South London and Maudsley NHS, OXLEAS, NELFT and SEPT NHS Foundation Trusts, the SLAM ‘Consent for Consent c4c’ initiative, with support of the Mental Health Research Network and via other research projects within the Psychosis Studies department at the Institute of Psychiatry, Psychology, Neuroscience (IoPPN), Kings College London. Controls were recruited through online announcements on local websites (e.g., Gumtree, Craigslist, Callforparticipants), via colleague researchers and circular emails for recruitment at the IoPPN.

***Supplement B****: Algorithm for partner responses in the trust game*

The first repayment was either 1.0, 1.1, 1.2, 1.3, 1.4 or 1.5 times the first investment, with an equal chance for returns of each factor. After the first trial, the factors were updated depending on the amount being invested compared to the amount invested in the previous trial, i.e., whether this was an increase or decrease. After an increase in trust or investment of the maximum amount (£10), an increment of 0.05 was added to each of the randomly selected six factors until each of the factors reached 1.6, reflecting higher repayments. Each factor would decrease with the same increments (-0.05) when trust decreased or remained at the minimum (£0), with a minimum value of 1 for each factor, reflecting lower repayments.

***Supplement C:*** *Whole-brain analyses: task effect*

Whole-brain analyses, over all participants, of activation during the cue phase revealed predominantly increased mPFC activity. The investment phase elicited activation in medial and lateral prefrontal and parietal regions. Activity during the repayment phase was increased within the medial and lateral prefrontal cortices, parietal regions and the caudate nucleus. These findings are in line with previous studies probing brain activity during the trust game (Bellucci et al., 2017; Fett et al., 2012a). No group, context or interaction effects were found. The overall effect of context over groups is shown below.

*
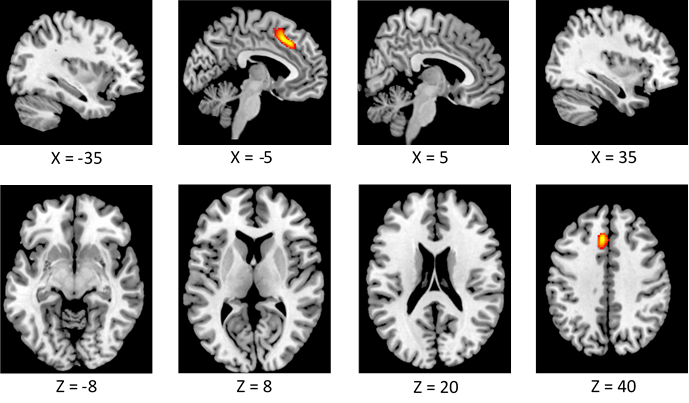
Cue phase in the trust game*

*Investment phase in the trust game*

*
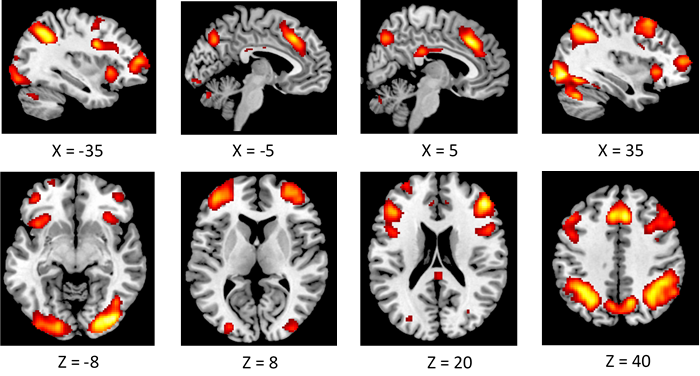
*

*
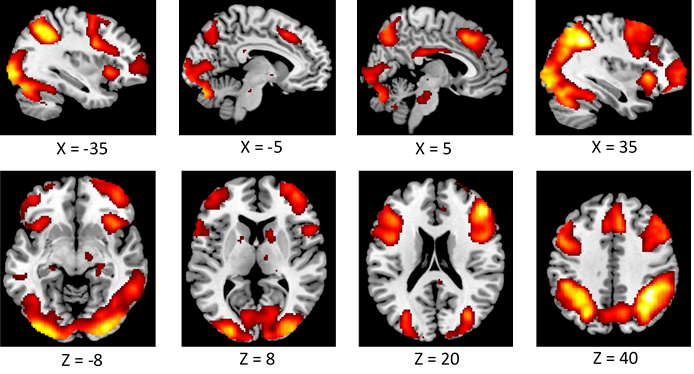
Repayment phase in the trust game*

***Supplement D:*** *ROI analyses over trials during the investment phase*

Given that we observed a behavioural group difference in investments over trials, we performed exploratory analyses probing the change in ROI activation over investment trials. We obtained a trial-by-trial estimates for the investment phase, by modelling the investment phase of one trial as regressor of interest, with all other trial phases and trials combined in one regressor of no interest in a trial specific general linear model. Subsequently, we contrasted each estimate of the experimental trials to the corresponding control trial. Next, for each ROI and each subject, we extracted the average signal change per trial to probe group and context effects on investment-related activation changes over trials. When inspecting the trial-by-trial estimates, there were large fluctuations in signal from trial-to-trial, suggesting unreliable estimates, although our trial-by-trial estimation was based on a widely used method^1^. There were no significant effects of group or trial number, or their interaction over trials in any ROI (all *p* > .09).

***Supplement E: Associations with symptoms***

For the analyses the PANSS positive scale score and PANSS negative scale scores (3-factor model) were used. We also ran exploratory analyses focussing specifically on paranoia (P6 item), and an amotivation factor (PANSS N2, N4 and G6) and diminished expression factor (PANSS N1, N3, N6 and G7) as previously used by Fervaha et al. (2014).

***First investments and symptoms.*** For first investments, there was no significant interaction with either the positive symptom scale, suspiciousness, negative symptom scale, amotivation, diminished expression and context, nor were there main effects of positive or negative symptoms, suspiciousness, amotivation or diminished expression on first investments (all *p* > .3).

***Investments over trials and symptoms.*** For investments over trials, there was a significant 3-way interaction between **positive symptoms**, context and trial number (*χ*^2^(2) = 6.02, *p* = .04). We did not find a significant interaction between positive symptoms and trial number in the negative or no context condition (*p* > .48), but there was a significant interaction in the positive context condition (*b* = .02, 95% CI [.007, .03], *p* = .001). To examine this interaction further we divided the patients into two groups by scores on the PANSS (low = average score of 1 and 2, high = score of 3 and up on the 7-point Likert-scale). We found that patients with low symptoms (N = 18) did show a decrease in investments (*b* = -.04, 95% CI [-.07, -.03], *p* < .001), whereas patients with high positive symptoms (N = 5) did not show changes in investments over trials (p = .58).

For **suspiciousness**, there was no significant 3-way interaction between context, trial number and suspiciousness (*p* =.55) and there were no two-way interactions (*p* =.11). The main effect of suspiciousness on investment was also not significant (*p* = .79).

For **negative symptoms,** there was a significant 3-way interaction (*χ*^2^(2) = 7.56, *p* = .02). Analyses by context did not show an interaction effect of negative symptoms and trial number or main effects of negative symptoms within the negative or the no context condition (all *p* > .06). However, we did find a significant interaction between negative symptoms and trial number in the positive context (*b* = .14, 95% CI [.009, .02], *p* < .001). To investigate this interaction further, we made two symptom groups (low = average score of 1 and 2, high = score of 3 and higher). The interaction was caused by slopes in opposing directions, with decreasing investments in patients with low negative symptoms (N = 13), (*b* = -.08, 95% CI [-.12, -.04], *p* < .001) and increasing investments in patients with high negative symptoms (N = 10), (*b* = .06, 95% CI [.01, .12], *p* = .013).

For **amotivation** there was a significant three-way interaction (*χ*^2^(2) = 8.23, *p* < .01). Analyses by context did not show an interaction effect of negative symptoms and trial number or main effects in the no context condition (all *p* > .58). However, we did find a significant interaction of negative symptoms and trial number in the positive (*b* = .06, 95% CI [.04, .09], *p* < .001) and the negative context condition (*b* = .02, 95% CI [-.003, .05], *p* = .53). To examine this interaction, we divided the patients into two groups by scores on the amotivation factor (low = average score of 1 and 2, high = score of 3 and higher). In the positive and negative context condition, there was a significant decline of investments in patients with low amotivation ((N = 15), *b* = -.06, 95% CI [-.09, -.04], *p* < .0001 and *b* = -.07, 95% CI [-.10, -.03], *p* < .0001), respectively). In patients with high amotivation (N = 8) there was a significant increase in investments (*b* = .07, 95% CI [.01, .12], *p* = .01) in the positive - and no significant effect in the negative context condition *(p = .*48). For **diminished expression** there was no significant interactions and no main effect (all *p >*.06).

***ROI activation and symptoms.*** On a neural level, we explored whether positive symptoms, level of suspiciousness, negative symptoms and amotivation and diminished expression were associated with activation in the ROIs that yielded significant group differences, i.e., the left dlPFC during context presentation and the right caudate during repayment. In case of significant interactions, analyses were conducted to investigate the association in patients with low and high symptoms (low = score of 1 and 2, high = score of 3 and up on the 7-point Likert-scale)

***DlPFC and symptoms.*** For **positive symptoms** there was a significant interaction with context on dlPFC activation (*χ*^2^(2) = 21.03, *p* <.0001). Analysis by group showed that for patients with low positive symptoms (N = 18) there was no difference in dlPFC activation in the no and positive context (*p* = .5) and that dlPFC activation was higher in the negative than the no context condition (*b* = .20, 95% CI [.09, .32], *p* < .001). For patients with high positive symptoms (N = 5) there was higher dlPFC activation in the negative and the positive condition compared to the no context condition (*b* = .26, 95% CI [.01, .05], *p* = .04) and (*b* = .46, 95% CI [.21, .71], *p* < .001, respectively).

For **suspiciousness**, there was no significant interaction between context and dlPFC activation (*p* =.78). There was a main effect of suspiciousness on dlPFC activation (*b* = .18, 95% CI [-.001, .036], *p* = .05).

There was a significant interaction between **negative symptoms** and context on dlPFC activation (*χ*^2^(2) = 112.34, *p* <.0001). In patients with low negative symptoms (N = 13) dlPFC activation was higher in the negative and positive context compared to the no context condition (*b* = .13, 95% CI [.004, .024], *p* = .04) and (*b* = .50, 95% CI [.38, .63], *p* < .001), respectively. In patients with high negative symptoms (N = 10) there was higher dlPFC activation in the negative compared to the no context condition (*b* = .34, 95% CI [.17, .50], *p* < .0001) and lower activation in the positive than the no context condition (*b* = -.50, 95% CI [-.66, -.33], *p* < .0001, respectively).

There was a significant interaction between **amotivation** and context on dlPFC activation (*χ*^2^(2) = 155.97, *p* <.0001). In patients with low amotivation (N = 15) there was higher dlPFC activation in the negative and positive compared to the no context condition (*b* = .44, 95% CI [.33, .57], *p* < .0001) and (*b* = .61, 95% CI [.49, .73], *p* = .009), respectively. In patients with high amotivation (N = 8) there was lower dlPFC activation in the negative and the positive condition compared to the no context condition (*b* = -.20, 95% CI [-.37, -.04], *p* = .001) and (*b* = -.94, 95% CI [-1.10, -.78], *p* < .0001, respectively).

There was a significant interaction between **diminished expression** and context on dlPFC activation (*χ*^2^(2) = 52.20, *p* <.0001). In patients with low diminished expression (N = 12) there was higher dlPFC activation in the negative and positive compared to the no context condition (*b* = .24, 95% CI [.11, .38], *p* < .0001) and (*b* = .36, 95% CI [.23, .50], *p* < .0001), respectively. In patients with high diminished expression (N = 11) there was higher dlPFC activation in the negative compared to the no context condition (*b* = .18, 95% CI [.03, .34], *p* = .02) and lower activation in the positive compared to the no context condition (*b* = -.26, 95% CI [-.41, -.09], *p* = .001).

***Caudate and symptoms.*** For **positive symptoms** there was a significant interaction with context on caudate activation (*χ*^2^(2) = 29.17, *p* <.0001). In patients with low positive symptoms (N = 18) there was no difference in caudate activation between the positive, negative and no context condition (*p* = .48). For patients with high positive symptoms (N = 5) there was lower caudate activation in the positive condition compared to the no context condition (*b* = -.24, 95% CI [-.30, -.18], *p* < .0001), but no difference in activation between the no context and negative context condition (*p* = .16).

For **suspiciousness**, there was a significant interaction with context on caudate activation (*χ*^2^(2) = 35.29 *p* <.0001). In patients with low suspiciousness (N = 10) there was no difference in caudate activation in the no and positive context (*p* = .12) and that caudate activation was higher in the negative than the no context condition (*b* = .11, 95% CI [.06, .16], *p* < .0001). In patients with high suspiciousness (N = 13) there was lower caudate activation in the negative and positive condition compared to the no context condition (*b* = -.11, 95% CI [-.15, -.07], *p* <.0001 and (*b* = -.10, 95% CI [-.14, -.06], *p* <.0001, respectively).

There was a significant interaction between **negative symptoms** and context on caudate activation (*χ*^2^(2) = 63.37, *p* <.0001). Patients with low negative symptoms (N = 13) showed no difference in caudate activation in the no and negative context (*p* = .5), but caudate activation was higher in the positive than the no context condition (*b* = .06, 95% CI [.05, .10], *p* = .009). Patients with high negative symptoms (N = 10) showed lower caudate activation in the negative and the positive- compared to the no context condition (*b* = -.05, 95% CI [-.09, -.01], *p* = .009 and *b* = -.17, 95% CI [-.21, -.13], *p* = .009, respectively).

There was a significant interaction between **amotivation** and context on caudate activation (*χ*^2^(2) = 69.41, *p* <.0001). In patients with low amotivation (N = 15) caudate activation was higher in the negative than the no context condition (*b* = .06, 95% CI [.02, .10], *p* = .002), but there was no difference between the positive and no context condition *(p* = .19)*.* In patients with high amotivation (N = 8) there was lower caudate activation in the negative and the positive condition compared to the no context condition (*b* = -.16, 95% CI [-.21, -.12], *p* < .0001 and (*b* = -.17, 95% CI [-.21, -.13], *p* < .0001, respectively).

There was a significant interaction between **diminished expression** and context on caudate activation (*χ*^2^(2) = 191.71, *p* <.0001). In patients with low diminished expression (N = 12) there was no difference in caudate activation in the no and negative context (*p* = .3), but caudate activation was higher in the positive than the no context condition (*b* = .08, 95% CI [.03, .13], *p* = .001). In patients with high diminished expression (N = 11) there was lower caudate activation in both the negative and the positive- compared to the no context condition (*b* = -.06, 95% CI [-.09, -.02], *p* = .002 and (*b* = -.18, 95% CI [-.22, -.14], *p* < .0001, respectively).

**References**

**1.** Mumford JA, Turner BO, Ashby FG, Poldrack RA. Deconvolving BOLD activation in event-related designs for multivoxel pattern classification analyses. *Neuroimage* 2012;59(3):2636-2643.

**2.** Brown RG, Pluck G. Negative symptoms: the ‘pathology’of motivation and goal-directed behaviour. *Trends in neurosciences* 2000;23(9):412-417.
